# Childhood Obesity: Prevalence and Prevention in Modern Society

**DOI:** 10.7759/cureus.31640

**Published:** 2022-11-18

**Authors:** Shambhavi Kumari, Samarth Shukla, Sourya Acharya

**Affiliations:** 1 Pathology, Jawaharlal Nehru Medical College, Datta Meghe Institute of Medical Sciences, Wardha, IND; 2 Medicine, Jawaharlal Nehru Medical College, Datta Meghe Institute of Medical Sciences, Wardha, IND

**Keywords:** coronary disease, diabetes, genetic, metabolic syndrome, malnutrition, hypertension, obesity

## Abstract

Over the previous few years, childhood obesity rates have risen globally. Obesity is defined as an accumulation of adipose tissue that is of sufficient magnitude to impair health. There is a significant negative impact of obesity on a child’s health both in childhood as well as in adulthood. Both industrialized and emerging nations face severe public health risks due to the increased prevalence of obesity in children. Overweight children are more likely to develop obesity as they get older. Children who are overweight struggle with physical exercise. Therefore, children should be encouraged to include any form of physical activity in their daily routine. Parents play a major role in adapting a child to a healthy environment. Assessing the child’s nutritional adequacy concerning what the child consumes and the recommended diet is vital. In some regions of the world, obesity has replaced malnutrition as the primary issue, with nutrition, overweight, and obesity being up to four times more prevalent than malnutrition. Worldwide, there have been significant changes in lifestyle over the last few years that have led to less physical activity and higher calorie-dense food intake. Obesity in children may lead to hypertension, coronary disease, and a greater incidence of diabetes complications and metabolic syndrome. It is necessary to develop new methods for treating and preventing childhood obesity. This article examines the widespread presence of childhood obesity, its various causes and consequences, as well as available interventions.

## Introduction and background

An excessive amount of body fat that is of sufficient magnitude to impair health is known as childhood obesity. It can be assumed that when caloric intake surpasses energy expenditure, obesity results [1]. The increasing prevalence of obesity globally appears to be significantly influenced by environmental variables, lifestyle choices, and cultural context [1]. However, the majority of the time, obesity is substantially influenced by individual lifestyle decisions and the culture of society [1]. Over the past 40 years, especially in the developed world, there has been an astounding increase in the percentage of children who are obese [2]. Still, developing nations are also seeing an increase in its occurrence. Childhood obesity is among the most serious public health concerns currently. The situation is pervasive and is beginning to impact many low and working-class regions, particularly in urban areas. The prevalence is rising at an alarming rate [2]. Childhood obesity is a risk factor for metabolic syndrome, poor physical health, psychological disorders, respiratory illnesses, and risk for diabetes. Developing countries like India have a particular issue known as a “double burden,” with starvation and malnutrition at one end of the spectrum and obesity at the other [2]. According to estimates from various studies, 200 million schoolchildren are either overweight or obese [3].

While obesity was uncommon in children long ago, it is now the most prevalent metabolic and nutritional disorder [4]. According to the WHO, obesity is one of the most underdiagnosed conditions with a significant impact on public health. The fifth-most important threat for both affluent and low-fatality emerging countries, according to the 2002 World Health Report, is being overweight [4]. Being overweight is a problem that has been made worse by our lifestyles, which include too much sedentary activity and packaged foodstuffs, as well as our inherent inclination to accumulate fat in reaction to insulin - obesity and being overweight raise the risk of illness and mortality [4,5]. For instance, pregnancy-related gestational diabetes and high birth weight in newborns are predicted by women being overweight and obese during pregnancy. Obesity in adulthood and insulin resistance are expected to increase birth weight. Being overweight as a child causes comorbidities that can last up to two decades longer in an individual and can significantly impact the development of several risk factors for adult diseases [5]. The answer to addressing the present obesity epidemic may be prevention. Managing overweight or obesity may involve primary prevention, secondary prevention, or prevention of weight rebound after weight loss, as well as avoiding further weight gain in obese people who cannot lose weight. Although over half of adults worldwide are obese, it can be challenging to lose fat once excessive weight has taken hold. Children should be prioritized as a demographic for prevention strategies. Overall, there is a pressing need to start preventing and treating childhood obesity [6].

## Review

Epidemiology around the world

A significant public health concern on a global scale is childhood obesity. The incidence is still low in some developing nations but is higher in western and industrialized countries. The occurrence of obesity is higher around 30-40% in South America and the eastern Mediterranean than in Europe (20-30%), Southeast Asia (10-20%), the Western Pacific (10-20%), and Africa (10-20%). According to studies, 155 million, or one in 10 school-going children, are obese. In many developing countries, especially in metropolitan areas and among those with high socioeconomic levels, overweight and obesity have risen sharply in recent years [7]. The possibility of obesity was significantly greater in high-income societies in China [8]. Russian rural areas had a higher frequency of obesity, but Chinese urban areas had a higher prevalence. Interestingly, obesity and overweight were higher among kids than teenagers in Russia and China, but not in the United States [8]. Numerous studies in India have revealed that 10-30% of teenagers are obese [9].

Factors causing childhood obesity

Leptin

Leptin is essential in maintaining a healthy balance of energy and preventing excessive energy storage. Leptin concentrations are linked to satiety, lipid oxidation, and higher energy expenditure, supporting this activity. Leptin relates more to a balance between energy intake and energy expenditure than to each factor on its own. Adipose tissue mass is a significant factor affecting plasma leptin concentrations in people; changes in body fat and food intake control human leptin mRNA and protein. Leptin has been found to correlate positively with body mass index and fat mass, negatively with fat-free mass, and is typically higher in obese children than in lean children [10]. The main risk factor for obesity is leptin resistance. Leptin resistance is caused by many variables, including inflammation and stress on the endoplasmic reticulum (ER). Leptin levels are unusually high in obese people. This is due to a condition known as “leptin resistance,” in which some obese individuals’ brains do not react to leptin, causing them to continue eating despite having adequate or excessive fat stores. The fat cells start producing more leptin as a result [11].

Insulin

The pancreatic islets of Langerhans cells secrete insulin, a peptide hormone that regulates carbohydrates, fatty acids, and protein metabolism, stimulating growth as well as cell division via its mitogenic effects. Insulin helps cells absorb glucose and keeps blood sugar levels normal. Childhood and adolescent obesity are frequently and seriously complicated by insulin resistance. A decline in insulin’s ability to promote glucose utilization by muscles and adipose tissue and to reduce hepatic glucose synthesis and output is known as insulin resistance. Additionally, it explains why insulin has less of an impact on the metabolism of proteins and lipids, as well as the gene expression and function of vascular endothelial cells. Additionally, insulin resistance has been linked to the emergence of poor glucose tolerance and type 2 diabetes mellitus in obese children and adolescents [12].

Ghrelin

Ghrelin functions as an endogenous ligand of the orphan G-protein coupled receptor. Ghrelin has numerous metabolic effects, including increasing hunger, growth hormone release, and fat storage. Ghrelin has an orexigenic impact by affecting the hypothalamic pathways that control appetite. At the same time, in the peripheral organs, such as the liver and pancreas, it stimulates the formation of fatty tissue and has a diabetogenic effect [13]. Body mass index and eating habits influence plasma ghrelin levels. Obese people and those with insulin resistance typically have lower ghrelin levels. Ghrelin secretion is decreased in obesity and may contribute to growth hormone hyposecretion [14].

Sweetened Beverages

Sugary drinks have also been examined as a potential risk factor for obesity. Contrary to common belief, sugary drinks do not just refer to soda but also include juice and other artificially sweetened beverages. It has been observed that there is a link between drinking sugary beverages and being overweight [15]. Sugary beverages can be consumed more quickly than food and are less filling. The relationship between genetic predisposition and obesity might be affected if these beverages are consumed more often. Children who drink sugar-sweetened beverages have twice the risk of obesity than their average classmates [15]. The consumption of sugary beverages was considerably higher in obese children than in normal-weight youngsters. These drinks offer more empty calories than 100% fruit juices, which is a worry because they have no nutritional value [15].

Fast-Food Consumption

It is believed that having a lot of fast-food options nearby increases the incidence of childhood obesity because it discourages people from practicing healthy eating habits and promotes exposure to places that serve unhealthy food, leading to compensatory consumption of unhealthy foods [16]. Consuming unhealthy food, which exposes children to massive portion sizes with excessive calories and glucose loading, may significantly increase the rates of overweight and obesity [17]. Many families, especially those with two working parents, choose these places because they are often favored by their children and are both accessible and reasonably priced [18].

Physical Inactivity

Sedentary behavior has the strongest correlation with obesity. Young children and teenagers now watch more television than ever [19]. Kids are getting involved in gadgets and computer and video gaming rather than playing outside [20]. There is a decline in physical activity, both before and after school. Children have become more reliant on automobiles rather than bicycles even for shorter distances. Children have fewer possibilities to be physically active if they reside in dangerous regions or do not have accessibility to walking paths that are well-lit and secure [21].

External Factors

**Figure 1:**
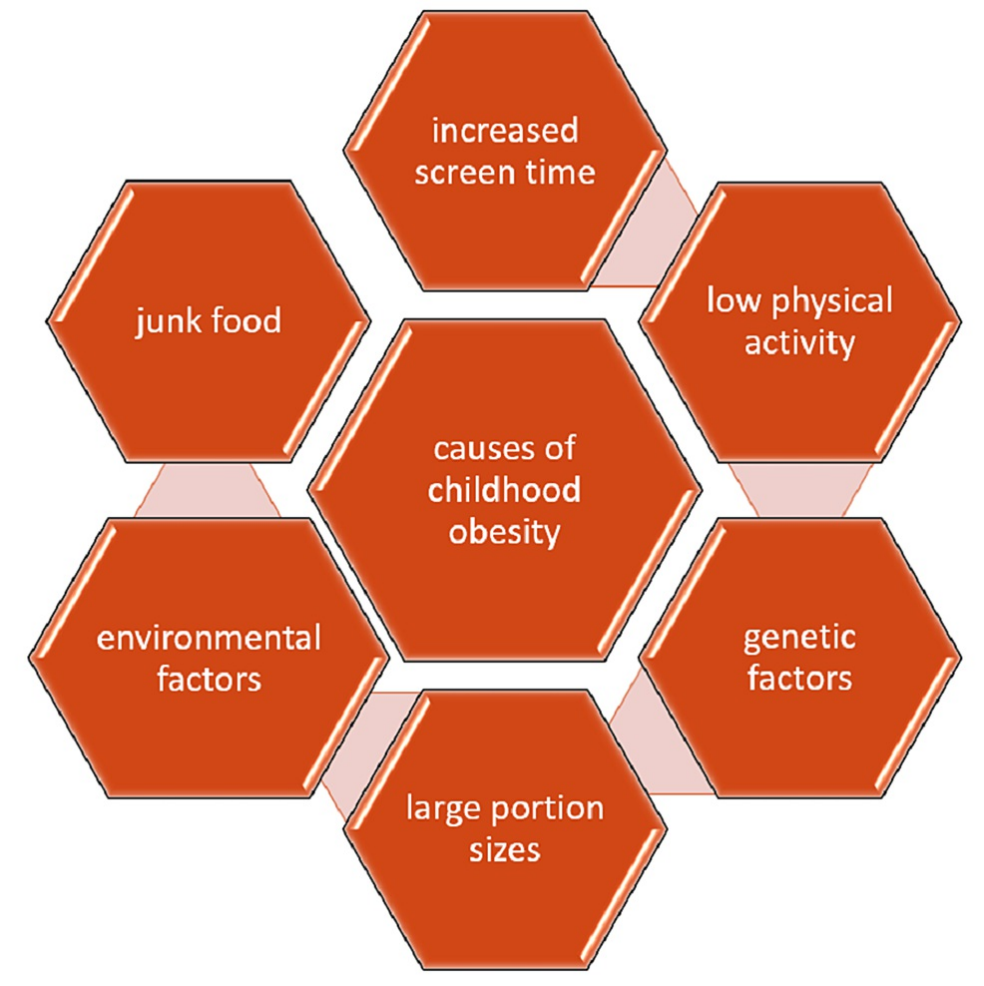
Causes of childhood obesity. Image created by the authors.

It has been noted that increasing town traffic congestion has resulted in several difficulties, such as sound, industrial air contamination, and other ecological difficulties, which can have an impact on children’s psychology, sleep, endocrine systems, and physical activity, and each have a connection to the obesity epidemic [22]. Children have been subjected to new and unfamiliar stressors due to widespread quarantine and house confinement during the coronavirus disease 2019 outbreak, which has increased childhood obesity [23]. Children’s emotional and physical state has been negatively impacted by school closings and subsequent home confinement measures as they no longer had access to any outdoor places for physical activity. Hence, the pandemic played a crucial role in recent years in childhood obesity [24]. Figure 1 shows the various causes of childhood obesity.

Effects of childhood obesity

The likelihood of metabolic syndrome is evident in Western countries due to obesity in children. Insulin resistance and undesirable body-fat patterning in metabolic syndrome may be caused by genetic predisposition or bad early-life experiences [25]. The prevalence of obesity nowadays significantly affects illness and mortality in the community; in this regard, the rising incidence of childhood obesity is of particular concern [26].

Children’s physical health, social and emotional well-being, and self-esteem can be significantly impacted by childhood obesity. Moreover, it is associated with a child’s lower standard of living and unsatisfactory academic performance [27]. Childhood obesity is frequently accompanied by comorbidities such as metabolic, cardiovascular, orthopedic, neurological, hepatic, pulmonary, and renal disorders [28].

Overweight children were more likely than their healthy-weight classmates to have several clinically significant linked psychosocial conditions, an increase in conduct problems such as disobedience, disruptive, aggressive and disastrous behavior, and physical and verbal abuse [29]. Other concerns include emotional symptoms, peer troubles, and focus problems [29]. Bullying and teasing are examples of the stigma attached to being overweight. Older obese children have a higher risk of developing depression and other internalizing illnesses, including anxiety and paranoia [30]. Children can develop non-alcoholic fatty liver disease, and as childhood obesity grows more common, this type of childhood liver disease is becoming more common [31].

Obese kids frequently experience menstrual irregularities. According to the theory that body weight and fatness are crucial physiologic triggers of menarche, obese girls are reported to experience menarche sooner, typically before the age of 10 [32]. In contrast, oligomenorrhoea or amenorrhoea is also linked to obesity because obese adolescent females typically develop polycystic ovarian syndrome and hyperandrogenism, which is again fuelled by insulin resistance brought on by visceral adiposity [33].

**Figure 2:**
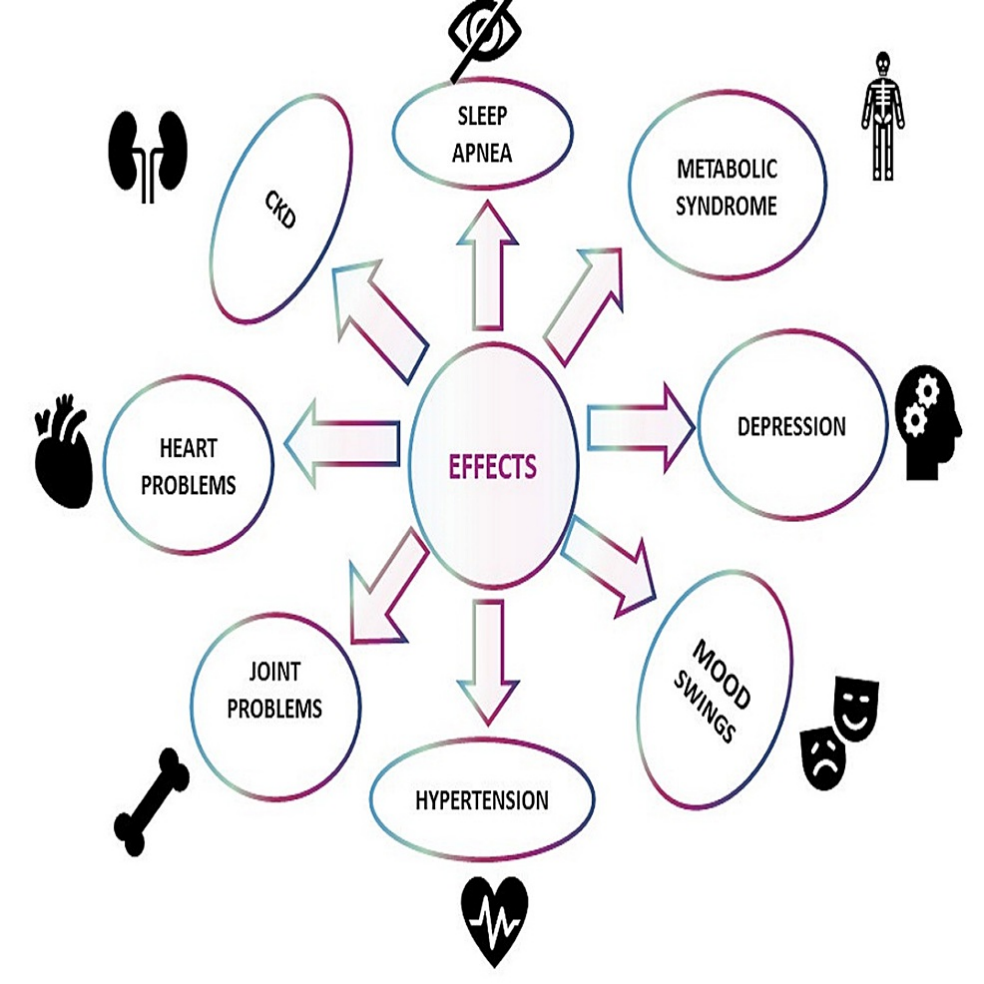
Effects of childhood obesity. Image created by the authors.

Childhood obesity is a crucial indicator of overall cardiovascular risk and the beginning of adult cardiovascular disease. Many childhood factors that lead to adult cardiovascular illness have been linked to the atherosclerotic process, which has been demonstrated to start in infancy. Dyslipidemia, hypertension, and insulin resistance are the cardiovascular outcomes of obesity [34]. Obesity in childhood can harm health outcomes later in life, increasing the risk of cancer, diabetes, depression, dyslipidemia, infertility, and cardiovascular disease mortality. Hence, being obese as a youngster could be a risk factor for adult morbidity [35]. Figure 2 shows the effects of childhood obesity.

Prevention

The prevalence of childhood obesity has significantly increased over the past few years and has become a severe public health issue. Because it is exceedingly difficult to treat once it has been established, prevention of childhood obesity through various forms of intervention seems to hold promise [36]. It appears that reduction in sedentary activities, family cooperation, and the promotion of physical exercise in conjunction with nutritional education may be crucial factors in preventing childhood obesity [37]. Strategies for changing dietary habits and exercise might be started at home, in preschool settings, schools, or after-school care facilities. The target population’s culture, ethnicity, as well as sociodemographic background should be taken into account when developing these tactics. Reduced intake of soft drinks, fast food, prepared snacks, and sugary foods may be helpful in stopping the rise in childhood obesity [38]. Interventions must fairly comprehend and consider the consequences of this attitude, especially given that many children are overweight, to promote the participation of the majority of children in sustained physical activity. In general, biological, sociocultural, and psychological factors, as well as how they interact, influence children’s physical exercise behavior. Most current research supports the idea that specific psychosocial characteristics, for instance, self-perception and physical competence, may serve as strong pillars for increasing the engagement of overweight and obese children in unstructured physical exercise. Therefore, interventionists should focus on enhancing these human attributes before addressing potential physiological and environmental causes. Children living with obesity may benefit from improving their motor abilities to feel better about themselves [39]. Table 1 lists the various ways to control childhood obesity.

**Table 1:** Various ways to control childhood obesity.

Serial number	Ways to control childhood obesity
1	Encourage physical exercise
2	Limit screen time
3	Ensure adequate sleep
4	Promote intake of fruits and vegetables
5	Avoid sugary and ultra-processed food items
6	Avoid mental stress
7	Check the nutritional content of the meal
8	Add fiber and protein to the diet
9	Preconception and pregnancy care

## Conclusions

The cause of childhood obesity is now understood to be an imbalance between energy intake and expenditure, and lifestyle choices and food preferences are strongly correlated with an improvement in positive energy balance. It is anticipated that childhood obesity rates will rise further. Therefore, it is crucial to consider its implications for physical and psychological health. The effects of childhood and adolescent obesity are extensive. They include not only physical health outcomes, such as high blood pressure, high cholesterol, metabolic syndrome, insulin resistance diabetes, orthopedic problems, sleep apnea, asthma, and hepatic steatosis, but also psychological, social, and behavioral outcomes, such as the increased risk for issues with body image, self-esteem, social exclusion and discrimination, depression, and a lower quality of life. To counteract this growing tendency that threatens the health and well-being of the younger generation and has a significant negative impact on resources and economic cost, a clear and strategic approach to combat the obesity epidemic is required by implementing comprehensive programs that encourage children and adolescents to eat healthy meals and decrease their consumption of unhealthy foods and beverages with added sugar. More focus is needed on these interventions in kids and teenagers. Parents should ensure that kids grow properly and form good habits, giving them advice and encouragement for a nutritious diet, enough sleep, and physical activity in the early years. Governments should develop and implement strategies that support active living among school-age children and adolescents, healthy school environments, nutrition and health knowledge, and multifaceted weight-management programs for adolescents and kids who are obese.
